# Polyphenol-Rich Extract of *Syzygium cumini* Leaf Dually Improves Peripheral Insulin Sensitivity and Pancreatic Islet Function in Monosodium L-Glutamate-Induced Obese Rats

**DOI:** 10.3389/fphar.2016.00048

**Published:** 2016-03-10

**Authors:** Jonas R. Sanches, Lucas M. França, Vinicyus T. Chagas, Renato S. Gaspar, Kayque A. dos Santos, Luciana M. Gonçalves, Deborah M. Sloboda, Alison C. Holloway, Richard P. Dutra, Everardo M. Carneiro, Ana Paula G. Cappelli, Antonio Marcus de A. Paes

**Affiliations:** ^1^Laboratory of Experimental Physiology, Department of Physiological Sciences, Federal University of MaranhãoSão Luís, Brazil; ^2^Laboratory of Endocrine Pancreas and Metabolism, Department of Estructural and Functional Biology, Institute of Biology, State University of CampinasCampinas, Brazil; ^3^Departments of Biochemistry, Pediatrics and Obstetrics and Gynecology, McMaster UniversityHamilton, ON, Canada; ^4^Department of Obstetrics and Gynecology, McMaster UniversityHamilton, ON, Canada; ^5^Social, Health and Technological Sciences Center, Federal University of MaranhãoImperatriz, Brazil

**Keywords:** metabolic syndrome, insulin resistance, hypertriglyceridemia, obesity, insulin secretion, jambolan, polyphenols, complementary and alternative medicine

## Abstract

*Syzygium cumini* (L.) Skeels (Myrtaceae) has been traditionally used to treat a number of illnesses. Ethnopharmacological studies have particularly addressed antidiabetic and metabolic-related effects of extracts prepared from its different parts, especially seed, and pulp-fruit, however. there is a lack of studies on phytochemical profile and biological properties of its leaf. As there is considerable interest in bioactive compounds to treat metabolic syndrome and its clustered risk factors, we sought to characterize the metabolic effects of hydroethanolic extract of *S. cumini* leaf (HESc) on lean and monosodium L-glutamate (MSG)-induced obese rats. HPLC-MS/MS characterization of the HESc polyphenolic profile, at 254 nm, identified 15 compounds pertaining to hydrolysable tannin and flavanol subclasses. At 60 days of age, both groups were randomly assigned to receive HESc (500 mg/kg) or vehicle for 30 days. At the end of treatment, obese+HESc exhibited significantly lower body weight gain, body mass index, and white adipose tissue mass, compared to obese rats receiving vehicle. Obese rats treated with HESc showed a twofold increase in lipolytic activity in the periepididymal fat pad, as well as, brought triglyceride levels in serum, liver and skeletal muscle back to levels close those found in lean animals. Furthermore, HESc also improved hyperinsulinemia and insulin resistance in obese+HESc rats, which resulted in partial reversal of glucose intolerance, as compared to obese rats. HESc had no effect in lean rats. Assessment of *ex vivo* glucose-stimulated insulin secretion showed HESc potentiated pancreatic function in islets isolated from both lean and obese rats treated with HESc. In addition, HESc (10–1000 μg/mL) increased glucose stimulated insulin secretion from both isolated rat islets and INS-1E β-cells. These data demonstrate that *S. cumini* leaf improved peripheral insulin sensitivity via stimulating/modulating β-cell insulin release, which was associated with improvements in metabolic outcomes in MSG-induced obese rats.

## Introduction

The occurrence of the Metabolic Syndrome (MetS) has reached epidemic proportions becoming a major socioeconomic issue globally, affecting countries regardless of human development index (HDI) values and ethnicity (O’Neill and O’Driscoll, 2015). MetS is consensually defined as the co-occurrence of three out of five possible dysfunctional characteristics: obesity/central obesity, insulin resistance (IR), fasting hyperglycemia, hypertension, and dyslipidemia, mainly observed as hypertriglyceridemia (Alberti et al., 2009). Given this cluster of modifiable outcomes, as well as their progressive pathophysiological changes, a variety of complementary and alternative medicinal approaches, such as practice of regular exercise, consumption of functional foods and traditional herbal preparations, have been adopted in addition to the pharmacological regimens commonly used at distinct stages of MetS (Rubio-Ruiz et al., 2013). In this scenario, herbal medicines have emerged as the leading source of bioactive molecules to target MetS because of their large-scale phytochemical diversity, which includes numerous flavonoids, and polyphenolic-related compounds (Arts and Hollman, 2005; Galleano et al., 2012; Song et al., 2014).

*Syzygium cumini* (L.) Skeels (Fam.: Myrtaceae; Syn.: *Syzygium jambolanum* (Lam.) DC, *Eugenia jambolana* Lam., *Eugenia cumini* (L.) Druce) has been used for centuries by Indian Ayurvedic medicine practitioners for diverse therapeutic purposes (Ayyanar et al., 2013). However, it was only in the mid to late nineteenth century that its particular capacity to decrease urine sugar content in diabetic patients impelled British traders to import it to Europe (Helmstadter, 2008). Thereafter, *S. cumini* became a worldwide cultivated medicinal plant, being popularly known as jamun in India, black plum in Europe, jambolan in Spanish-spoken countries, and jambolão in Brazil (Ayyanar and Subash-Babu, 2012). The antidiabetic and other metabolic-related properties have been investigated specially using the fruit and seeds, whereas the leaves have been underused and under investigated (Ayyanar et al., 2013). The antihyperglycemic impact of seed extracts has been demonstrated in diabetic animal models (Ravi et al., 2004; Sharma et al., 2006, 2008). Administration of aqueous seed extract (400 mg/Kg/day, 21 days) to high-fat diet/streptozotocin-induced diabetic rats reduced IR via upregulation of hepatic peroxisome proliferators-activated receptors (PPAR-α and -γ) (Sharma et al., 2012). Meanwhile, other metabolic-related properties such as anti-hyperlipidemic and cardioprotective activities have been attributed to insulin-independent mechanisms, e.g., inhibition of 3-hydroxy-3-methylglutaryl-coenzyme A (HMG-CoA) reductase by ethanolic seed kernel extract (100 mg/Kg/day, 30 days) (Ravi et al., 2005) as well as reduction of atherosclerotic apolipoprotein B100 synthesis by a fraction from aqueous fruit pulp extract (20 mg/Kg/day, 30 days) (Tanwar et al., 2011) in streptozotocin-induced diabetic rats. In a recent work, we reviewed the main secondary metabolities that have been reported in different parts of *S. cumini*, especially flavonoids and polyphenolic-related compounds, correlating them to the potential mechanisms of action that might underlie the aforementioned pharmacological properties (Chagas et al., 2015).

Most investigations of the metabolic properties of *S. cumini* have been carried out in animals presenting with aggressive hyperglycemia and elevated oxidative stress derived from alloxan- or streptozotocin-induced diabetes, quite distinct from the usual manifestations of human MetS. Animal models of mild to moderate MetS, including the well-validated monosodium L-glutamate (MSG)-induced obesity model (Miranda et al., 2014) offer a more relevant opportunity to further examine the pharmacological properties of *S. cumini*. In this model, the administration of MSG to newborn rats result in damage to the hypothalamic nuclei, particularly the median eminence and arcuate nucleus, leading to impaired growth hormone (GH) pulsatile secretion and autonomic nervous system unbalance characterized by exacerbated parasympathetic tonus. At adulthood, MSG-obese rats present with stunted growth, abdominal obesity, hyperinsulinemia, IR, impaired glucose tolerance, and dyslipidemia (Olney, 1969; Millard et al., 1982; Miranda et al., 2014). We have recently shown that MSG-induced dyslipidemia is mainly characterized by hypertriglyceridemia subsequent to early activation of endoplasmic reticulum (ER)-stress and IR in the liver (Franca et al., 2014).

In the present study, we took advantage of the prediabetic MSG-obesity model to further investigate the metabolic properties of the hydroethanolic extract of *S. cumini* leaf (HESc) in rats. Here we show that phytochemical characterization of HESc by HPLC-MS/MS mainly consists of hydrolysable tannins and flavanols, which confer it an elevated antioxidant capacity. After 30-days of oral treatment with HESc, obese rats exhibited slower weight gain, abdominal fat mass loss, serum and peripheral-tissue lipid profiles amelioration, and attenuation of glucose intolerance, associated with the recovery of peripheral insulin sensitivity and improvement of pancreatic islet function. Lean HESc-treated rats did not show any significant changes, although demonstrated increased insulin release. This observation is consistent with a dual role for HESc, and its polyphenolic compounds, as a stimulator/modulator of insulin-glucose axis homeostasis in MSG-induced obese rats.

## Results and Discussion

### Antioxidant Capacity and HPLC-MS/MS Characterization of HESc Polyphenolic Profile

**FIGURE 1:**
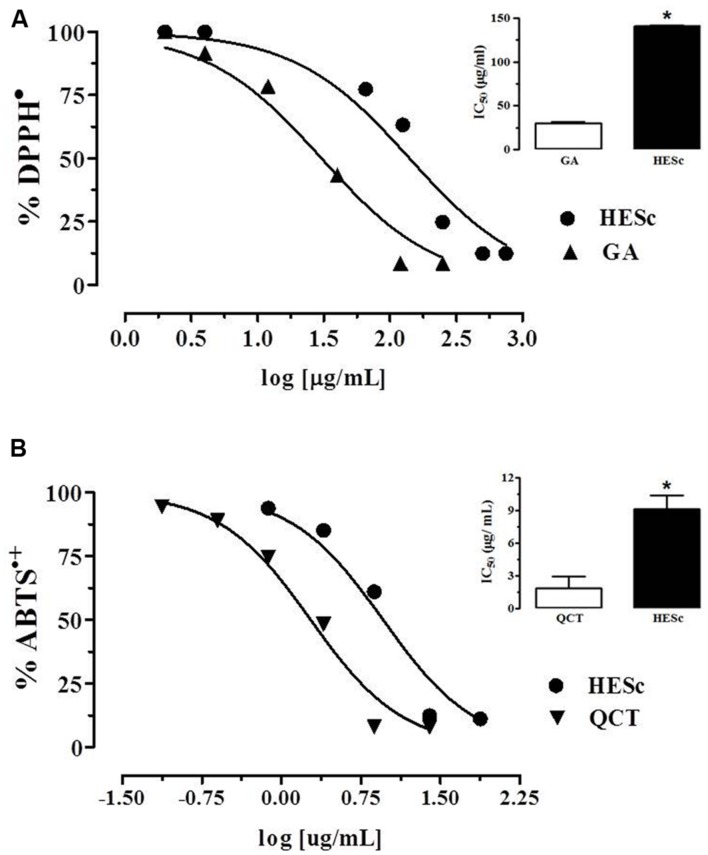
**Antioxidant capacity of hydroethanolic extract of *Syzygium cumini* leaf (HESc).** Antioxidant capacity of HESc against the radicals **(A)** 1,1-diphenyl-2-picrylhydrazyl (DPPH^•^) and **(B)** 2,2′-azino-bis-3-ethylbenzthiazoline-6-sulphonic acid (ABTS^•+^). Inserts represent inhibitory concentration 50% (IC_50_) for HESc and standards gallic acid (GA) and quercetin (QCT). Data represent mean or mean ± SEM from experiments run in quadruplicate. ^∗^*p* < 0.05 analyzed by Student’s test.

Flavonoids and polyphenolic-related compounds, abundant in fruits, and vegetables are used for dietary and medicinal purposes, and increasing evidence supports the beneficial effects of such compounds on gluco-lipid homeostasis and the prevention of metabolic diseases (Arts and Hollman, 2005). Thus, we first assessed polyphenolic content and *in vitro* antioxidant capacity of HESc. Prussian Blue method was applied to determine total polyphenol content in HESc, which resulted in 23.0 ± 1.1 GAE/100 g. This polyphenolic content is higher than those described in extracts prepared from other parts of the plant, such as seed (Arun et al., 2011) and fruit (Veber et al., 2015), but lower than those previously described to the leaf (Ruan et al., 2008; Veber et al., 2015). However, in these works, values would have been superstimated since authors applied Folin–Ciacoteau method, which also quantifies non-phenolic compounds like aromatic aminoacids, sugars, and organic acids, making it inadvisable for total polyphenol content quantifications (Prior et al., 2005). Assessment of antioxidant capacity showed that HESc efficiently scavengers both 2,2-diphenyl-1-picrylhydrazyl (DPPH^•^) and 2,2’-azino-bis(3-ethylbenzothiazoline-6-sulphonic acid) (ABTS^•+^) radicals (**Figure 1**). When compared to antioxidant standards, HESc ability to scavenger DPPH was 4.5 times less than gallic acid (GA) (HESc IC_50_ 140.80 ± 1.20 μg/mL vs. GA IC_50_ 30.5 ± 1.2 μg/mL, insert in **Figure 1A**), whereas it showed higher potency against ABTS^•+^, as compared to quercetin (HESc IC_50_ 9.1 ± 1.2 μg/mL vs. QCT 1.9 ± 1.2 μg/mL, insert in **Figure 1B**). Similar findings have been shown for *S. cumini* leaf extracts, but at higher inhibitory concentrations (Ruan et al., 2008; Jagetia et al., 2012). Our data further support biological appliance of HESc, since ABTS^•+^ method correlates with other antioxidant assays that simulate physiological radical reactions, as well as, properly depict the activity of polyphenols and flavonoids contained in fruits, vegetables and their derivatives (Floegel et al., 2011).

**FIGURE 2:**
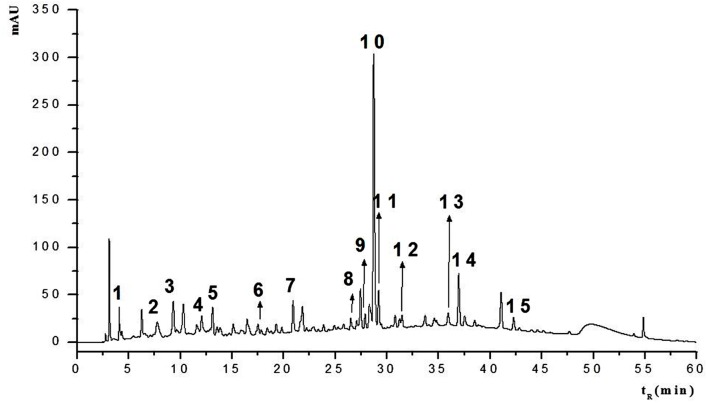
**HPLC fingerprint of the hydroethanolic extract of *S. cumini* leaf (HESc).** HPLC chromatogram with UV detection at 254 nm of hydroethanolic extract of *S. cumini* leaf (HESc). Peak numbers correspond to tentatively identified compounds denoted in **Table 1**.

HPLC-MS/MS was used to determine HESc polyphenolic profile. **Figure 2** shows the HPLC chromatogram, recorded at 254 nm. The information from the spectral data (Supplementary Figure S1) was compared with published data to allow the tentative identification of fifteen compounds (peaks numbered 1–15 in **Figure 2**) pertaining to hydrolyzable tannins (ellagitannins and gallotannins) and flavonols. **Table 1** lists all the compounds identified with their chromatographic retention time and mass spectral data. Compound **1** was identified as hexahydroxydiphenoyl-glucose (HHDP-glucose), whose [M–H]^-^ at *m/z* 481 fragmented to yield daughter ions at *m/z* 301 and 275. Compounds **2, 3, and 5** showed [M–H]^-^ at *m/z* 783 and fragments at *m/z* 481, 301, and 275, which were identified as isomers of di-HHDP-glucose, also known as pedunculagin I (Gordon et al., 2011; Santos et al., 2011; Tala et al., 2013). Compound **6** showed an [M–H]^-^ at *m/z* 935, which yielded a fragment at *m/z* 633 due to the loss of one unit of ellagic acid (*m/z* 301). This compound was identified as di-HHDP-galloyl-glucose, also known as casuarinin (Simirgiotis and Schmeda-Hirschmann, 2010; Fischer et al., 2011). Compound **4**, which presented [M–H]^-^ at *m/z* 761 with fragments at *m/z* 635 and 609, was identified as (*epi*) gallocatechin-(*epi*)gallocatechin-*O*-gallate (Tala et al., 2013). Compounds **7**, **8**, **9**, **10,** and **12** were identified as galloyl monosaccharides, which resulted from esterification of glucose by 3–5 GA units (galloyl, *m/z* 170). Interestingly, it has been shown that the number of galloyl units determine antioxidant capacity of galloylglucose isomers (Ngoc et al., 2008). Peaks **11**, **13**, **14,** and **15** were tentatively identified as myricetin-derived compounds (Gordon et al., 2011). Compound **11** was identified as myricetin deoxyhexoside due to the loss of 146 Da from [M–H]^-^ at *m/z* 463 to MS^2^ at *m/z* 317. Compounds **13** and **14** were identified as isomers of acylated myricetin deoxyhexoside since the decrease of [M–H]^-^ from *m/z* 505 to MS^2^ at *m/z* 463 may be ascribed to the loss of an acyl group (*m/z* 42). Finally, compound **15**, whose [M–H]^-^ at *m/z* 519 fragmented to yield MS^2^ at *m/z* 477, 331, and 315, was identified as acylated methylmyricetin deoxyhexoside, once the loss of 188 Da might correspond to an acylated hexose unit (42 + 146 Da).

**Table 1:** Tentative identification of polyphenolic profile of the hydroethanolic extract of *Syzygium cumini* leaves (HESc).

Peak	t_R_ (min)	Compound	[M–H]^-^ (*m/z*)	MS^2^ (*m/z*)	Reference
1	4.1	HHDP-glucose	481	301, 275	Gordon et al., 2011
2	7.9	Di-HHDP-glucose	783	481, 301, 275	Gordon et al., 2011; Santos et al., 2011; Tala et al., 2013
3	9.4	Di-HHDP-glucose	783	481, 301, 275	Gordon et al., 2011; Santos et al., 2011; Tala et al., 2013
4	11.8	(*epi*)gallocatechin-(*epi*)gallocatechin-*O*-gallate	761	635, 609, 593	Tala et al., 2013
5	13.2	Di-HHDP- glucose	783	481, 301, 275	Gordon et al., 2011; Santos et al., 2011; Tala et al., 2013
6	17.5	Di-HHDP-galloylglucose	935	633	Santos et al., 2011; Wu et al., 2012
7	21.0	Trigalloylglucose	635	465	Pfundstein et al., 2010; Gordon et al., 2011
8	26.7	Tetragalloylglucose	787	635, 617, 447	Gordon et al., 2011
9	27.7	Tetragalloylglucose	787	635, 617, 447	Gordon et al., 2011
10	28.4	Tetragalloylglucose	787	635, 617, 447	Gordon et al., 2011
11	29.2	Myricetin deoxyhexoside	463	317	Gordon et al., 2011
12	31.6	Pentagalloylglucose	939	769, 617	Gordon et al., 2011
13	35.9	Acylated myricetin deoxyhexoside	505	463, 316	Gordon et al., 2011
14	37	Acylated myricetin deoxyhexoside	505	463, 316	Gordon et al., 2011
15	42.3	Acylated methylmyricetin deoxyhexoside	519	477, 331, 315	Gordon et al., 2011

*HHDP, hexahydroxydiphenoyl.*

Previous work has shown *S. cumini* leaf to be rich in acylated flavonol glycosides, especially myricetin, myricitrin, quercetin, kaempferol, in addition to simple phenols, like ellagic acid, ferulic acid, and GA (Ruan et al., 2008; Ayyanar and Subash-Babu, 2012). In our work, 4 out of 15 compounds identified in HESc were myricetin-derived flavonols whose occurrence have been already reported in *S. cumini* fruits (Gordon et al., 2011) and leaves (Mahmoud et al., 2001). Myricetin and its derivatives have been shown to improve insulin signaling pathways in skeletal muscles (Tzeng et al., 2011) and adipocytes (Wang et al., 2015), as well as, to protect pancreatic β-cells from cytokine-induced cell death (Ding et al., 2012). It is noteworthy that ellagitannins (compounds **1**–**3**, **5,** and **6**) and gallotannins (compounds **7**–**10** and **12**) were identified in our HESc, since these compounds have been previously described only in jambolan pulp-fruit (Gordon et al., 2011; Aqil et al., 2012). Antioxidant properties of these ellagitannins are well documented (Fischer et al., 2011; Kahkonen et al., 2012); for instance, casuarinin has been shown to inhibit adipocyte differentiation, as well as, to promote insulin-like effects (Bai et al., 2008). The (*epi*)gallocatechins are important proanthocyanidins with inhibitory activity against α-amylase, a particularly interesting property for diabetes treatment (Wang et al., 2012). Compound **4**, which was tentatively identified as an epigallocatechin gallate derivative, has been shown to improve insulin secretion and protect pancreatic β-cells from glucotoxicity (Cai and Lin, 2009). To the best of our knowledge, this is the first time that flavan-3-ols like epigallocatechin gallate has been found in *S. cumini* leaf. Overall, the polyphenolic profile herein described for HESc not only ensures our extract genuineness, but also strengthens our pursuit for the mechanisms of action underlying the important biological properties that are reported in this work.

### HESc Improves Morphometric Parameters and Attenuates Weight Gain in Obese Rats

We sought to investigate *in vivo* and *ex vivo* effects of oral chronic administration of HESc on morphometric and metabolic profiles of lean and MSG-obese rats. Since there are no published pharmacokinetic studies available for *S. cumini* extracts, we took into consideration parameters established for other plant species (Lei et al., 2003; Li et al., 2008) to estimate a dose of 500 mg/kg/day to allow secondary metabolites present in HESc to reach biologically relevant steady state plasma concentrations. Notwithstanding, polyphenolic compounds, e.g., flavonols and procyanidins, are kept stable during gastric transit, although factors like gut microflora or specific tissue perfusion rate might interfere on their bioavailability at biological targets (Fraga and Oteiza, 2011).

**FIGURE 3:**
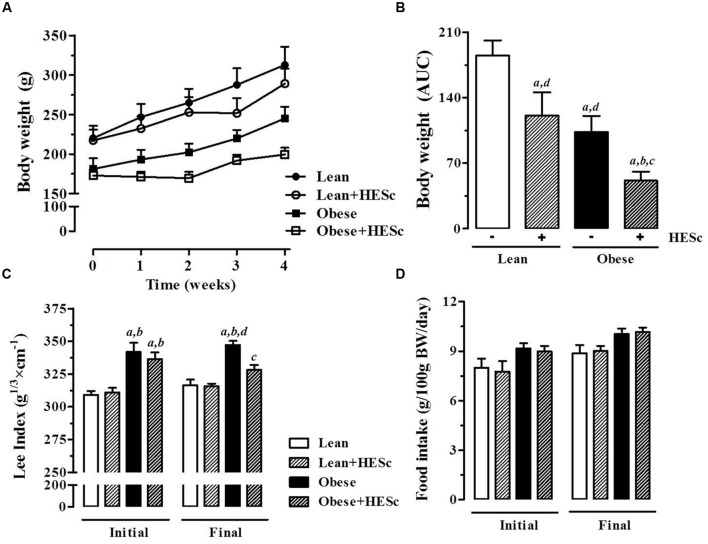
**Effect of hydroethanolic extract of *S. cumini* leaf (HESc) on morphometric parameters.** 60 days-old lean and obese rats were treated or not with HESc (500 mg/kg/day, v.o.) for 30 days. **(A)** Body weight evolution and **(B)** area under curve (AUC) are shown for all groups. Initial (day 0) and final (day 30) values for **(C)** Lee Index [body weight (g)^1/3^/naso-anal length (cm) × 100]; and **(D)** food intake are also shown. Results are expressed as mean ± SEM (*n* = 6–8 per group). Letters indicate significant difference (*p* < 0.05) against ^a^Lean; ^b^Lean+HESc; ^c^Obese; or ^d^Obese+HESc, analyzed by one-way ANOVA followed by Newman–Keuls post-test.

Consistent with previous reports, prior to HESc treatment, obese rats were lighter, despite having higher Lee Index and similar daily food consumption, compared to lean controls (**Figure 3**) (Olney, 1969; Millard et al., 1982). After 30-days of treatment, HESc-treated obese rats were ∼60% lighter (173 ± 7 to 199 ± 9 g) than obese rats without treatment (181 ± 13 to 245 ± 15 g) (*p* < 0.05 **Figure 3A**). Calculation of area under body weight curve showed HESc prevented weight gain in both lean and obese rats (**Figure 3B**), whereas no difference was observed in the final body weight between lean groups (**Figure 3A**). HESc also decreased the Lee Index from 336 ± 5 to 328 ± 4 in MSG+HESc rats (**Figure 3C**), despite no changes in food intake (**Figure 3D**). Lee Index is a surrogate formula to assess rodent body mass, especially in MSG-obese rodents, which show stunted growth (Bernardis and Patterson, 1968), but is not very useful for dietary or developmental obese models (Stephens, 1980). Additionally, HESc consistently decreased white adipose tissue depots in obese+HESc rats, with no effect on lean controls (**Table 2**). HESc had no effect on brown adipose tissue, skeletal muscle or liver relative masses (**Table 2**).

**Table 2:** Morphological parameters and lipid profiles of insulin-sensitive tissues upon HESc 30-days treatment of lean and MSG-obese rats.

	Lean	Lean+HESc	MSG	MSG+HESc
**Morphological parameters (g/100g BW)**				
Retroperitoneal fat	0.70 ± 0.15	0.60 ± 0.11	3.59 ± 0.20^a,b,d^	2.60 ± 0.17^a,b,c^
Periepididymal fat	0.70 ± 0.14	0.64 ± 0.07	4.63 ± 0.28^a,b,d^	2.91 ± 0.21^a,b,c^
Mesenteric fat	0.62 ± 0.10	0.57 ± 0.09	2.73 ± 0.15^a,b,d^	2.24 ± 0.25^a,b,c^
Interscapular brown fat	0.07 ± 0.002	0.07 ± 0.003	0.17 ± 0.02^a,b^	0.16 ± 0.01^a,b^
Liver	3.3 ± 0.3	2.9 ± 0.1	2.5 ± 0.1^a^	2.5 ± 0.1^a^
Soleus muscle	0.04 ± 0.002	0.04 ± 0.002	0.03 ± 0.002^a,b^	0.03 ± 0.002
Gastrocnemius muscle	0.55 ± 0.03	0.59 ± 0.01	0.45 ± 0.04	0.45 ± 0.03
**Liver lipid profile(mg/g)**				
Total fat	185 ± 19	197 ± 19	267 ± 19^a,b,d^	187 ± 17^c^
Triglycerides	5.9 ± 0.7	6.9 ± 0.3	18.4 ± 2.4^a,b,d^	8.7 ± 1.0^c^
Total cholesterol	2.6 ± 0.2	3.0 ± 0.3	2.8 ± 0.4	2.5 ± 0.2
**Skeletal muscle lipid profile (mg/g)**				
Total fat	259 ± 13	283 ± 26	259 ± 11	268 ± 25
Triglycerides	4.4 ± 0.6	5.2 ± 1.0	13.2 ± 1.4^a,b,d^	8.0 ± 1.0^c^
Total cholesterol	1.4 ± 0.1	1.5 ± 0.2	1.3 ± 0.1	1.3 ± 0.1

*Data represent mean ± SEM (n = 6–8), comparison between group pairs were performed by ANOVA, using Newman–Keuls as post-test. Superscript letters indicate statistical difference (p < 0.05) against ^a^Lean, ^b^Lean+HESc, ^c^MSG, ^d^MSG+HESc. Tissues were collected upon euthanasia performed after 12-h fasting.*

**FIGURE 4:**
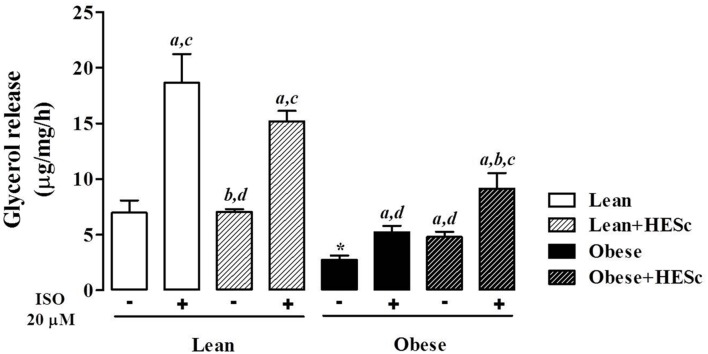
**Effect of hydroethanolic extract of *S. cumini* leaf (HESc) on lipolytic activity of periepididymal white adipose tissue.** Basal (-) and 20 μM isoproterenol (ISO)-induced lipolytic activity was assessed in peridepididymal white adipose tissue samples collected from 90 days-old lean and obese rats treated or not with HESc (500 mg/kg/day, v.o.) for 30 days. Results are expressed as mean ± SEM (*n* = 6–8 per group). Letters indicate intra-group significant difference (*p* < 0.05) against ^a^basal (-); ^b^ISO (+); ^c^HESc (-); or ^d^HESc/ISO (+), analyzed by one-way ANOVA followed by Newman–Keuls post-test. ^∗^*p* < 0.05 Lean basal (-) vs. Obese basal (-) inter-group analysis.

To further characterize HESc effects on adiposity, we measured basal and isoproterenol-evoked *ex vivo* lipolysis in periepididymal fat pads from all groups. Basal unstimulated lipolysis was increased 2-fold in obese+HESc rats, as compared to MSG controls (**Figure 4**; 2.7 ± 0.4 vs. 4.8 ± 0.4 μg/mg/h, *p* < 0.05); this level of lipolysis in the HESc-treated obese rats approached the levels seen in lean rats (7.0 ± 1.1 μg/mg/h). Upon sympathetic stimulus with 20 μM isoproterenol, lipolytic activity showed very similar increases between HESc-treated and not treated rats from the same group, suggesting HESc had no further adrenergic impacts (**Figure 4**). Of note, obese rats, treated or not, presented lower lipolytic activity (**Figure 4**) that could be attributed to the low sympathetic activity already described for MSG obesity model (Martins et al., 2004). It has been shown that a flavonoid-rich extract from *S. cumini* seed (100 μg/mL, 8 days) upregulated both PPAR-α and PPAR-γ in 3T3-L1 preadipocytes (Sharma et al., 2008), which may improve adipocyte homeostasis leading to lower fat accumulation *in vivo*. Furthermore, 100 μM myricetin (Wang et al., 2015) and 10 μM epigallocatechin gallate (Chan et al., 2011) have been shown to enhance lipolysis and downregulate mRNA levels of adipogenesis-related transcription factors in the same 3T3-L1 preadipocytes. Considering the variety of myricetin derivatives found in HESc, besides other polyphenols, it is plausible that the anti-obesity properties herein described for *S. cumini* leaf may be due similar mechanisms, although this remains to be confirmed.

### HESc Improves Triglyceride Levels in Serum, Liver, and Skeletal Muscle of Obese Rats

**FIGURE 5:**
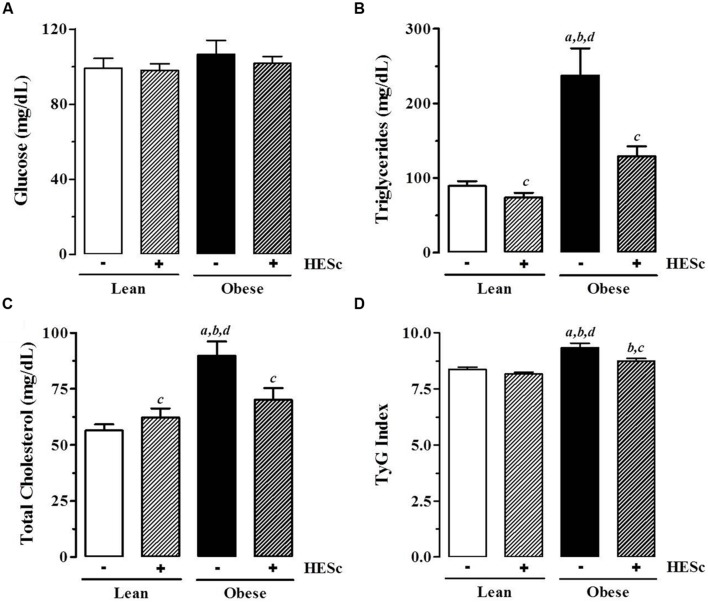
**Effect of hydroethanolic extract of *S. cumini* leaf (HESc) on serum biochemical parameters.** Serum levels of glucose **(A)**, triglycerides **(B)**, total cholesterol **(C)** were measured in blood samples collected from overnight fasting 90 days-old Lean and Obese rats treated or not with HESc (500 mg/kg/day, v.o.) for 30 days. **(D)** TyG Index was calculated from fasting glucose and triglyceride levels to infer insulin resistence. Results are expressed as mean ± SEM (*n* = 6–8 per group). Letters indicate significant difference (*p* < 0.05) against ^a^Lean; ^b^Lean+HESc; ^c^Obese; or ^d^Obese+HESc, analyzed by one-way ANOVA followed by Newman–Keuls post-test.

Concomitant with the effects on white adipose tissue accumulation, HESc also improved the serum biochemical profile of obese animals. Overnight fasting serum glucose levels were not changed by HESc administration in either lean or obese rats (**Figure 5A**); although serum triglyceride levels were strongly decreased in HESc-treated obese rats by 45% from 237 ± 36 mg/dL (obese) to 129 ± 13 mg/dL (obese+HESc); the HESc-treated obese rats had serum triglycerides levels that were not different from lean rats (lean 89 ± 7 mg/dL, lean+HESc 74 ± 6 mg/dL, **Figure 5B**). Total serum cholesterol levels were also reduced by 20% in obese+HESc rats (70 ± 5 mg/dL), as compared to obese rats (89 ± 6 mg/dL) (*p* < 0.05 **Figure 5C**). Hepatic as well as skeletal muscle triglyceride accumulation were ∼threefold higher in obese versus lean rats, but treatment with HESc normalized these levels found in lean animals. There were no significant differences in total cholesterol content in liver or skeletal muscle between any treatment groups (**Table 2**). Since triglyceride-rich VLDL particle overproduction is tightly associated with hepatic IR (Kamagate et al., 2008), we calculated TyG Index as a surrogate measure of hepatic insulin sensitivity (Simental-Mendia et al., 2008). HESc partially restored insulin sensitivity of obese rats, as TyG values were decreased from 9.5 ± 0.1 (obese) to 8.7 ± 0.1 (obese+HESc, *p* < 0.05, **Figure 5D**), although it was still different from lean control rats (lean 8.4 ± 0.1, lean+HESc 8.2 ± 0.1, *p* < 0.05). TyG Index is well validated for IR prediction in humans (Vasques et al., 2011; Du et al., 2014), but has also been increasingly applied for animal studies as well (Gonzalez-Torres et al., 2015; Motta et al., 2015). Even though we have not measured gene or protein expression of hepatic insulin signaling pathway markers, constituting a limitation in this work, our data strongly suggest that HESc targeted triglyceride biosynthetic pathways through mechanisms that might involve the improvement of insulin-dependent actions.

In a recent work, we showed that hypertriglyceridemia observed in MSG-obese rats is associated to activation of ER-stress pathways and increased expression of microsomal triglyceride-transfer protein (MTP) in the liver, resulting in higher VLDL assembly (Franca et al., 2014). MTP gene expression is controlled by transcription factor *forkhead box O1* (FoxO1) phosphorylation, which is reduced under insulin resistant conditions (Kamagate et al., 2008). Regarding the anti-dyslipidemic properties of *S. cumini*, it has been particularly demonstrated that ethanolic extracts from seed (100 mg/Kg/day, 21 days) or seed kernel (100 mg/Kg/day, 30 days) inhibit HMG-CoA reductase, the rate-limiting enzyme of cholesterol biosynthesis (Ravi et al., 2005; Sharma et al., 2008). However, our data showed that HESc completely reverted triglyceride accumulation in bloodstream and insulin-sensitive tissues, specifically liver, and skeletal muscle, of obese+HESc rats, which led us to speculate that HESc might improve peripheral insulin sensitivity. Indeed, the effects of HESc on hepatic and serum lipid profiles resemble those described for green tea (–)-epigallocatechin-3-gallate, which were attributed to a modulatory effect on lipogenic overexpressed genes of mice fed a high-fat diet (Lee et al., 2009). In high fat-fed rats, treatment with myricetin (300 mg/Kg/day, 8 weeks) resulted in significant lowering of plasma and hepatic triglycerides accumulation, an effect ascribed to overexpression of PPARα-related enzymes, which decreases the intracellular levels of fatty acids available for triglyceride synthesis in response to decreased IR (Chang et al., 2012). Similar effects have been described for *S. cumini* extracts (Sharma et al., 2012) and further support hepatic IR amelioration as the leading pathway by which *S. cumini* and its metabolites restore VLDL assembly and serum triglyceride levels in this study.

### HESc Restores Insulin-Glucose Homeostasis in MSG-Obese Rats

**FIGURE 6:**
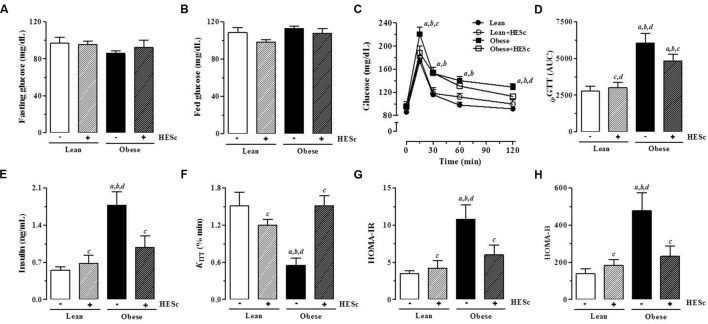
**Effect of hydroethanolic extract of *S. cumini* leaf (HESc) on insulin-glucose axis function.** Intraperitoneal glucose (*ip*GTT) and insulin (*ip*ITT) tolerance tests were performed to examine insulin-glucose axis function in 90 days-old Lean and Obese rats treated or not with HESc (500 mg/kg/day, v.o.) for 30 days. **(A)** Fasting and **(B)** fed serum glucose levels were measured in blood drops collected by tail-cut method. **(C)** Changes in tail blood glucose during *ip*GTT and **(D)** total blood glucose accumulation reported as AUC. **(E)** Fasting plasma insulin levels. Insulin sensitivity was assessed by **(F)** *K*_ITT_, which measure glucose disappearence rate from bloodstream and **(G)** HOMA-IR, homeostasis model index for insulin resistance (IR). **(H)** HOMA-B, homeostasis model index for β-cell function. Results are expressed as mean ± SEM (*n* = 6–8 per group). Letters indicate significant difference (*p* < 0.05) against ^a^Lean; ^b^Lean+HESc; ^c^Obese; or ^d^Obese+HESc, analyzed by one-way ANOVA followed by Newman–Keuls post-test.

In order to further investigate the effects of HESc on insulin-glucose axis function, all animals were subjected to intraperitoneal glucose (*ip*GTT) and insulin (*ip*ITT) tolerance tests at the end of treatment, with a 3-days gap between tests for proper volemic recovery. Baseline tail vein blood samples were taken for measurement of fasting and fed glucose levels, as well as, fasting insulin levels. There were no differences in fasting or fed serum glucose levels between any groups (**Figures 6A,B**, respectively). Upon intraperitoneal glucose loading for *ip*GTT, all groups reached maximal glucose levels within 15 min. HESc brought glucose levels at peak (obese+HESc, 188 ± 12 mg/dL) back to values near those of lean rats, while obese rats reached mean values 220 ± 12 mg/dL at the peak, persisting at higher levels throughout the test (**Figure 6C**, *p* < 0.05). Calculation of *ip*GTT area under curve (AUC) showed that glucose tolerance was partially restored in obese+HESc animals (**Figure 6D**), achieving intermediate values between lean and obese rats. **Figure 6E** shows that fasting insulin levels in obese rats were ∼threefold higher than lean controls (lean, 0.55 ± 0.06; lean+HESc, 0.68 ± 0.14; obese, 1.76 ± 0.25 ng/mL; *p* < 0.05), whereas HESc decreased insulin levels in obese rats by 42% (obese+HESc rats 0.98 ± 0.22 ng/mL; *p* < 0.05). Consistent with these data, calculation of *K*_ITT_ showed that insulin sensitivity was completely restored in obese+HESc rats, while obese rats presented values as low as half the other groups (**Figure 6F**). These results were further confirmed by assessment of HOMA-IR Index, which showed that peripheral insulin sensitivity in obese+HESc rats was completely restored and identical to lean rats (**Figure 6G**). Moreover, calculation of HOMA-B Index further suggested that HESc might interfere in the well-described insulin oversecretion of MSG-obese rats (**Figure 6H**).

The MSG model is an animal model of prediabetic metabolic syndrome, in which animals do not necessarily exhibit dysglycemia or hyperglycemia, despite being hyperinsulinemic, insulin-resistant, dyslipidemic, and abdominally obese (Olney, 1969). In fact, it has been observed MSG-obese rats as old as 6-months to present lower glycemia when compared to lean controls (Liu et al., 2011). Thus, facing the normoglycemic status of our rats, the absence of a hypoglycemiant effect on HESc-treated rats should not be considered a limitation in this study, rather it is in accordance with previous observations that glucose lowering properties of *S. cumini* constituents are more effective under hyper- than normoglycemic conditions (Ayyanar et al., 2013). Antidiabetic properties of *S. cumini* have been extensively studied since the mid-19th century, but no precise mechanistic action for any of its secondary metabolites has been proposed (Helmstadter, 2008; Ayyanar et al., 2013). Our data suggest that HESc was able to improve glucose tolerance as a result of increases in insulin sensitivity. These results are consistent with a previous report in which aqueous extract of *S. cumini* seeds (400 mg/Kg/day, 21 days) was found to improve insulin sensitivity through modulation of hepatic PPARγ downstream pathways (Sharma et al., 2012), an effect also described for a flavonoid-rich extract from *S. cumini* seeds on 3T3-L1 preadipocytes (Sharma et al., 2008). Considering leaf properties, methanolic extract of leaves (100 ng/mL) was found to increase mRNA expression of GLUT-4 glucose transporter and phosphatidylinositol-3 kinase (PI3 kinase) in L6 myotubes, both important mediators of insulin action in adipocytes and skeletal muscle (Anandharajan et al., 2006). Of note, very similar effects have been described for myricetin (Liu et al., 2007; Chang et al., 2012), which is highly prevalent in our HESc extract (**Table 2**). Finally, fasting serum insulin levels of obese+HESc rats were decreased to nearly 50% of those found in obese rats, leading us to hypothesize that abovementioned improvement of peripheral metabolic status might also exert a role on insulin secretion. Free fatty acids directly stimulate insulin release through activation of G protein-coupled receptor 40 (GPR40) on pancreatic β-cell surface (Itoh et al., 2003). Although we did not measure circulating levels of free fatty acids, a limitation in this report, obese+HESc rats showed strong decrease of triglyceride levels (**Figure 5B**; **Table 2**), which may lead to lower GPR40 activation and consequent decrease of insulin secretion. Our data are further supported by recent findings that methanol extract of *Baccharis dracunculifolia* (400 mg/Kg/day, 30 days) decreased serum insulin levels in MSG-obese rats subsequent to improved peripheral insulin sensitivity (Hocayen et al., 2015), in spite of insulin oversecretion caused by high parasympathetic activity on pancreatic islets of MSG-obese rodents (Miranda et al., 2014). Authors attributed this effect to the high antioxidant capacity of the extract (Hocayen et al., 2015), a property well-demonstrated by HESc.

### *Ex Vivo* Insulin Release is Increased in Isolated Pancreatic Islets from HESc-Treated Lean and Obese Rats

**FIGURE 7:**
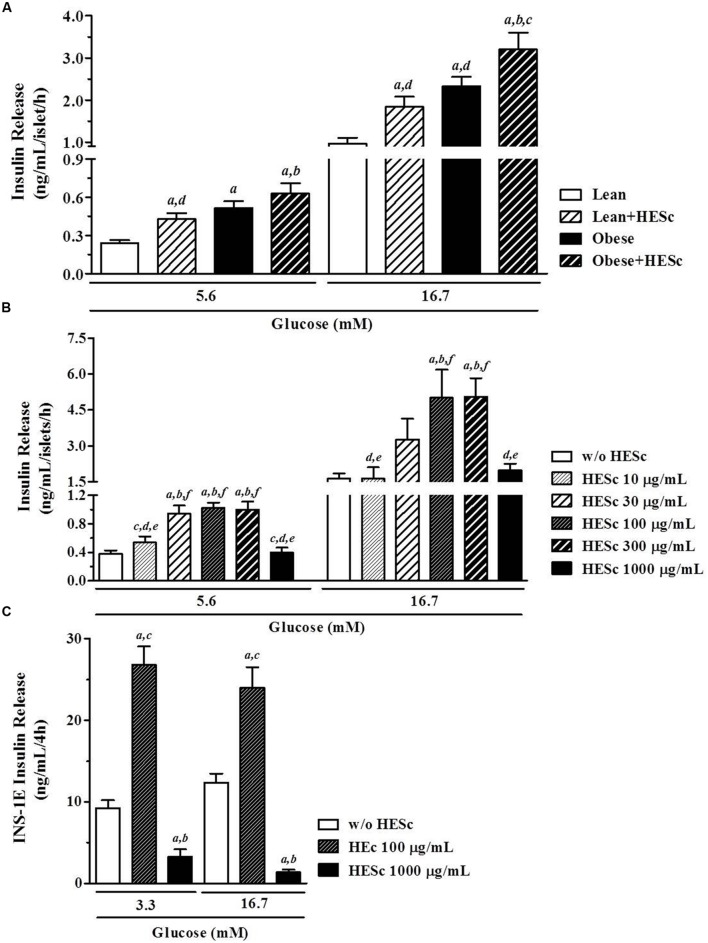
**Effect of hydroethanolic extract of *S. cumini* leaf (HESc) on *ex vivo* and *in vitro* glucose-stimulated insulin secretion (GSIS). (A)** GSIS (5.6 and 16.7 mM glucose) in pancreatic islets isolated from 90 days-old Lean and Obese rats treated or not with HESc (500 mg/kg/day, v.o.) for 30 days. **(B)** GSIS (5.6 and 16.7 mM glucose) in pancreatic islets isolated from control not-treated rats and incubated with increasing concentrations of HESc (10–1000 μg/mL). In both **(A,B)**, at least four groups of four islets per animal were incubated for 1 h at 37°C under denoted conditions. **(C)** GSIS (3.3 and 16.7 mM glucose) in INS-1E β-cells (2 × 10^5^ cells/well) incubated with 100 and 1000 μg/mL HESc for 4 h at 37°C. Results are expressed as mean ± SEM (*n* = 6–8 per group). In **(A)**, letters indicate significant difference (*p* < 0.05) against ^a^Lean; ^b^Lean+HESc; ^c^Obese; or ^d^Obese+HESc. In **(B)**, letters indicate significant difference (*p* < 0.05) against basal (a, w/o HESc) or 10–1000 μg/mL HESc (b–f, respectively). In **(C)**, letters indicate significant difference (*p* < 0.05) against basal (a, w/o HESc) or 100 and 1000 μg/mL HESc (**B,C**, respectively). All data were analyzed by one-way ANOVA followed by Newman–Keuls post-test.

We next assessed the effect of 30-days HESc treatment on glucose-stimulated insulin secretion in pancreatic islets isolated from lean and obese rats. Sets of 4 islets, in a minimum of four sets per animal, were incubated under basal (5.6 mM glucose) or stimulating (16.7 mM glucose) conditions for 1 h at 37°C. Basal insulin release was almost 80% higher in lean+HESc (0.43 ± 0.05 ng/mL/h; **Figure 7A**) than lean rats (0.24 ± 0.02 ng/mL/h, *p* < 0.05); but no significant increase was observed in obese+HESc rats over their obese counterparts. With increased glucose, insulin secretion in lean+HESc (1.84 ± 0.24 ng/mL/h) kept a similar elevation of 90% over lean controls (0.96 ± 0.14 ng/mL/h; **Figure 7A**, *p* < 0.05). Meanwhile, insulin release from islets isolated from obese+HESc rats (3.20 ± 0.39 ng/mL/h) was only 37% higher than that of obese animals (2.32 ± 0.22 ng/mL/h; **Figure 7A**, *p* < 0.05). The lower proportional increase of glucose-stimulated insulin release in obese+HESc rats allows us to suggest that pancreatic islets of MSG-obese rats are exhausted or have a potential impairment on the biochemical pathway by which HESc is promoting its secretagogue effect. Notably, pancreatic islets from all groups showed similar four to fivefold increase in insulin release with glucose stimulation, suggesting there must be some preserved secretory responsiveness to glucose (**Figure 7A**).

Glucose is the major physiological stimulator of insulin stimulus-secretion coupling, since it promotes an increase of ATP/ADP ratio with consequent closure of ATP-sensitive K^+^ channels (K^+^_ATP_), β-cell membrane depolarization and Ca^2+^ influx that triggers insulin-containing granules exocytosis (Sweet et al., 2004). However, glucose also elicits insulin secretion by way of K^+^_ATP_ channel-independent pathways (Gembal et al., 1993). It has been shown that adult MSG-obese rats have preserved functioning of the K^+^_ATP_ channel-dependent pathway despite showing dysfunctional K^+^_ATP_ channel-independent pathways. It has been speculated that glycolitic metabolism is enhanced in β-cells from MSG-obese rats, resulting in a lower threshold for glucose-stimulated insulin secretion (Grassiolli et al., 2006). Indeed, our data are consistent with this, since isolated islets from obese and obese+HESc rats showed increased insulin secretion at both 5.6 and 16.7 mM glucose, compared to lean controls. Concerning insulinotropic effects of *S. cumini* extracts, early reports demonstrated *in vivo* and *in vitro* insulin releasing properties exerted by fruit-pulp and seed extracts, emphasizing they were more pronounced on pancreatic islets from normoglycemic than hyperglycemic streptozotocin-induced diabetic rats (Achrekar et al., 1991). More recently, it was shown that a partially purified fraction from fruit-pulp (25 mg/Kg/day, 7 or 15 days) totally restored glucose-stimulated insulin release in alloxan-induced diabetic rabbits by way of a hypothesized sulphonylurea-like mechanism of action (Sharma et al., 2006). Our data do not support this hypothesis, since islet insulin release from HESc-treated did not change their sensitivity to glucose compared to untreated rats, diminishing the possibility of an additional effect on K^+^_ATP_ channel-dependent mechanisms. On the other hand, the insulinagogue effect of dietary flavonoids is well documented (Fraga and Oteiza, 2011) and might support the effects herein presented for HESc. For instance, catechin-derivatives like (*epi*)gallocatechin-(*epi*)gallocatechin-*O*-gallate, which is present in our HESc extract (**Table 1**), were shown to promote insulin secretion by improving β-cell mitochondrial function, in addition to other effects like antioxidant, antiinflammatory, and glucotoxicity prevention (Cai and Lin, 2009). Nevertheless, HESc-treated obese rats had reduced serum insulin levels (**Figure 6E**), in spite of a higher *ex vivo* insulin release response under hyperglycemic conditions (**Figure 7A**), very similar to the effects of *Baccharis dracunculifolia* methanol extract on MSG-obese rats (Hocayen et al., 2015). This particular set of data is suggestive of a role for HESc as an activator of peripheral pathways evoked either by recovery of peripheral insulin sensitivity (**Figure 6F**), or by lowering of serum lipids (**Figure 5**), which may promote decreased *in vivo* insulin release in our obese+HESc rats.

### HESc Directly Promotes *In Vitro* Insulin Release in Pancreatic Islets and a β-Cell Line

To further assess the insulinogogue capacity of HESc, we incubated pancreatic islets isolated from lean normoglycemic rats with increasing concentrations of HESc, from 10 to 1000 μg/mL. Isolated islets showed a fourfold increase in glucose-estimulated insulin secretion when glucose concentration was increased from 5.6 to 16.7 mM (**Figure 7B**). Thereafter, HESc was added for 1 h to the incubation medium under both glucose conditions. Increasing concentrations of HESc improved insulin secretion in a bell-shaped manner with maximal response at 100 and 300 μg/mL (**Figure 7B**). No effect was seen under either the lowest or the highest concentrations of HESc, suggesting that HESc constituents probably act more as modulators than stimulators of insulin secretion. Similarly our data on *ex vivo* insulin secretion (**Figure 7A**), direct incubation of isolated islets with HESc did not potentialize the glucose-evoked fourfold increase of insulin secretion under 16.7 mM glucose, further suggesting a modulatory role for the secondary metabolites indentified in HESc.

Although insulin-secreting β-cells are the main cellular component of islets, it has been shown that non-β-cells, i.e., α- δ- and pancreatic polypeptide cells, are crucial components of insulin secretion homeostasis (Brereton et al., 2015). Thus, to examine whether HESc targets β-cell machinery, HESc was also incubated with rat insulinoma-derived INS-1E cells, which is a well-characterized model for studies on β-cell function (Merglen et al., 2004). Incubation of 100 μg/mL HESc in INS-1E cells doubled the insulin secretion for both glucose concentrations (**Figure 7C**), in spite of the fact that the cells failed to show increased insulin release under 16.7 mM glucose, which constitutes a limiting aspect in our study but already observed by others (Merglen et al., 2004). It has been shown that quercetin, a flavonoid already described in *S. cumini* leaf (Ruan et al., 2008) but not identified in our HESc phytochemical characterization, promotes insulin secretion by direct activation of L-type calcium channel in INS-1E cells (Bardy et al., 2013). A similar L-type calcium channel-activating effect was described for myricetin (Fusi et al., 2003), whose derivatives were identified in our HESc extract. Thus, we speculate that any or even some of the compounds contained in HESc may act in a similar way, either isolatedly or collectively within a phytocomplex.

The unexpected decrease of insulin secretion caused by 1000 μg/mL HESc in our cells, led us to assess its possible cytotoxicity over INS-1E cells. Trypan blue and BrdU assays were used and showed that HESc resulted in significant cell death only at this concentration (data not shown). This is consistent with a previously reported antiproliferative effect of hydroethanolic extracts (12.5–200 μg/mL, 72 h) from fruit pulp or seed of *S. cumini* on lung cancer cells (Aqil et al., 2012), although myricetin has been described to protect RIN-m5f β-cells from cytokine-induced cell death (Ding et al., 2012). Since islet basal insulin secretion was maintained under this same HESc concentration in islet cultures (**Figure 7B**), we speculate that islet architecture may help to protect β-cells from the HESc cytotoxicity seen in INS-1E cells. This is consistent with the observation that a purified fraction isolated from *S. cumini* seeds (8 mg/Kg/day, 21 days) induced regenerative islets neoformation in streptozotocin-induced diabetic mice (Dusane and Joshi, 2011). Despite the abovementioned cytotoxic effect in INS-1E cells, hydroethanolic extract of *S. cumini* leaf, at dosis as high as 2 g/Kg, has been shown to be free of toxicity when chronically administered by oral route to rodents (Silva et al., 2012). Futhermore, one must recognize the possible metabolism of HESc compounds as a result of the first-pass through the liver potentially creating other unknown metabolites and need to be addressed in future pharmacokinetic studies.

## Conclusion

Although we have prioritized functional instead of molecular approaches in this study, considering the polyphenolic profile and the antioxidant capacity it confers to HESc, we speculate that the mechanisms whereby HESc acts appears to involve two pathways. The first is an improving effect on peripherally disrupted insulin action, supported by our observations of weight gain slowdown and effective IR overcome, as assessed in *K*_ITT_, higher lipolytic activity, reversal of triglycerides accumulation and improved glucose tolerance. The second pathway, likely to be synergistic with the first; may be a direct stimulatory/modulatory effect on pancreatic islet function, which disregards local glucose levels but probably involve the restoration of β-cell cytosolic redox status. To the best of our knowledge, it is the first time an insulinagogue effect is reported for *S. cumini* leaf. In conclusion, data presented in this study demonstrate the pharmacological relevance of *S. cumini* leaves as a potential source of bioactive compounds for the treatment of MetS and its associated comorbidities, especially presenting glucose intolerance, IR and/or pancreatic islet failure.

## Materials and Methods

### Plant Material and Preparation of Hydroethanolic Extract

Fresh leaves of *Syzygium cumini* (L.) Skeels were collected from different trees at Bacanga’s Campus of the Federal University of Maranhão (2°33′11.7″S 44°18′22.7″W), in São Luís, Maranhão, Brazil. Plant species authentication was performed by Prof. Dr. Eduardo Bezerra Almeida Jr., botanist of MAR – Herbarium of Maranhão – where a voucher specimen is deposited under #4574. Leaves were dried at 38°C in air-flow oven before pulverization and maceration in 70% ethanol (1: 6, w/v) for 3 days (solvent was replaced every 24 h) under continuous stirring at room temperature. Collected extract was evaporated under reduced pressure and lyophilized to yield the hydroethanolic extract of *S. cumini* leaf (HESc). HESc aliquots were kept at 4°C, protected from light, until further experimental use, when powdered HESc was resuspended in distilled water at desired concentrations.

### Assessment of Total Polyphenolic Content and Antioxidant Capacity

Prussian Blue method was applied for determination of total polyphenolic content in HESc, as previously described (Pueyo and Calvo, 2009). GA was used as standard and results expressed as GA equivalents per 100 g dry extract (GAE/100 g). Antioxidant capacity of HESc was assessed through 2,2′-azino-bis-3-ethylbenzthiazoline-6-sulphonic acid (ABTS^•+^) and 1,1-diphenyl-2-picrylhydrazyl (DPPH^•^) assays, as previously described (Floegel et al., 2011), using quercetin and GA as standards, respectively. All assays were performed in quadruplicate using freshly made solutions of HESc and standards.

### HPLC-MS/MS Analysis

Analysis were performed by HPLC-MS/MS using a chromatograph LC-20A Prominence (Shimadzu, Japan) equipped with autoinjector and detector SPD-20A/UV-Vis. HPLC separation was conducted on Luna C-18 analytical column (250 mm × 4.6 mm, 5 μm, Phenomenex), at a flow rate of 1 mL/min. Gradient elution was performed by using 0.1% formic acid acidified H_2_O (A) and acetonitrile (B), as mobile phases. Elution was performed according to the following conditions: 0–1 min 5% B; 1–30 min, 5–30% B; 30–60 min, 30–70% B, at 40°C and fingerprints detected at 254 nm. All solvents were purchased from Merck Millipore Co. (Germany) and degassed using ultrasonic bath. For analysis, HESc samples were dissolved in 70% EtOH (100 μg/mL) and filtered through 0.45 μm mesh (Millipore) for an injection volume of 25 μL. For mass spectra acquirement, chromatograph was coupled to an Esquire 3000 Plus mass spectrometer (Bruker Daltonics, Germany) with quadrupole ion trap analyzer in tandem mode with electrospray ionization (ESI). Ionization settings: ESI source voltage 40 V, potential 4 kV, temperature 320°C, ultrapure helium (He) as collision gas and nitrogen (N_2_) as nebulizer gas. The desolvation was facilitated using a current flow of 7.0 L/min. Data were acquired in full scan mode MS^2^ with negative ionization in the range of *m/z* 100–3000. Data on retention time, mass charge ratio (*m/z*) molecular ion and fragments were compared with available data in the literature to tentatively identification of phenolic compounds.

### Neonatal Induction of Obesity and Treatment with HESc

Newborn male Wistar rats (*Rattus norvegicus*, eight pups per dam) were subcutaneously injected with MSG (4 g/kg; obese group, Sigma–Aldrich) or equimolar saline solution (0.1 mL/10g, lean group) during the first 5 days of life from postnatal day 2–6) (Olney, 1969). After weaning (at postnatal day 21), animals were weighed twice a week to assess weight gain and kept under controlled conditions [RT 23 ± 2°C, 12 h light/dark cycle, filtered water and standard rodent chow (Nuvilab CR-1, Brazil) *ad libitum*]. At age 60 days of age, obesity was assessed by the calculation of the Lee Index, which is the quotient of the cube root of body weight (g) per naso-anal length (cm) (Bernardis and Patterson, 1968). All MSG-treated rats presented a Lee Index value significantly higher than lean controls mean. Thereafter, animals from MSG-treated and lean groups were randomly allocated into four groups for treatment with HESc or saline for 30 consecutive days, as follow:

**Lean**, lean rats receiving 0.1 mL/kg/day saline by gavage (*n* = 6).
**Lean+HESc**, lean rats receiving 500 mg/kg/day by gavage (*n* = 7).
**Obese**, MSG rats receiving 0.1 mL/kg/day saline by gavage (*n* = 8).
**Obese+HESc**, MSG rats receiving 500 mg/kg/day by gavage (*n* = 8).

During the whole treatment period, HESc solutions were freshly prepared by dissolving the lyophilized extract in saline. Animals and not-eaten chow were weighed every 2 days for body weight and food intake record. On treatment days 27 and 30, animals were submitted to glucose and insulin tolerance tests, respectively, as described later. After 30 days of treatment, Lee Index was once more performed to assess the effect of treatment on obesity. After overnight fasting, on day 31, the animals were anesthetized (10 mg/kg xylazine, 40 mg/kg ketamine) and subjected to retro-orbital bleeding for blood sample collection and were killed by exsanguination. Pancreas, liver, gastrocnemius, and soleus muscles, as well as periepididymal, retroperitoneal, visceral fat pads and interscapular brown adipose tissue were collected and weighed for morphometric assessment and further analyses. Morphometric data were expressed as tissue mass (g) per 100 g body weight (%BW, g). All protocols were approved by the Committee for Ethics and Welfare on Animal Use – CEUA of the Federal University of Maranhão under ruling N° 016/13.

### Serum Biochemistry Assessment

Blood samples were collected into EDTA and non-EDTA tubes to allow separation of both plasma and serum from all groups. Serum was used for spectrophotometric measurement of glucose, triglycerides and total cholesterol levels using commercial kits according to manufacturer’s instructions (Labtest, Brazil). Plasma was used for insulin measurement by radioimmunoassay (RIA) using human insulin radiolabelled with ^125^I as tracer, rat insulin as standard (Crystal Chem Inc.,USA) and rat insulin antibody (donated by Dr Leclerq-Meyer, Free University of Brussels, Belgium), according to previously described protocol (Scott et al., 1981).

### Liver and Skeletal Muscle Fat Extraction and Measurement

Samples of gastrocnemius and liver (∼500 mg each) were homogenized in 5 mL chloroform/methanol (2:1) solution, and kept overnight at 4°C. The resultant supernatant was filtered, added to 0.9% NaCl (saline 1:5 filtrate), stirred and left to stand for 2 h. Methanol and chloroform phases were separated by centrifugation (1000 rpm, 5 min) and a 1 mL aliquot of chloroform phase was collected and dried at 40°C in air-flow oven. Dry-fat was weighed and resuspended in 1 mL Triton-X100/methanol (2:1) for spectrophotometric measurement of triglycerides and total cholesterol levels using commercial kits according to manufacturer’s instructions (Labtest, Brazil). Results were expressed as total fat (mg) per tissue mass (g), triglycerides (mg) per tissue mass (g), and total cholesterol (mg) per tissue mass (g) (Freedman et al., 2005).

### *Ex Vivo* Lipolytic Activity Assessment

Samples of periepididymal white adipose tissue (∼100 mg) was shredded into small fragments and incubated in Krebs buffer (120 mM NaCl; 15 mM NaHCO_3_; 4.83 mM KCl; 1.2 mM MgSO_4_; 1.21 mM KH_2_PO_4_; 2.4 mM CaCl_2_, 1% BSA, and 0.1% glucose after pH adjustment to 7.4) under pumping aeration for 1 h at 37°C. For lipolytic activity assessment, incubation of samples was undergone in absence (basal) or presence of 20 μM isoproterenol (Sigma–Aldrich, USA). Reactions were stopped on ice bath and glycerol concentration on supernatant assessed by spectrophotometric measurement using an triglyceride commercial kit according to manufacturer’s instructions (Labtest, Brazil) (Vaughan, 1962).

### Intraperitoneal Glucose and Insulin Tolerance Tests

For intraperitoneal glucose tolerance test (*ip*GTT), animals were submitted to 8-h fasting prior to administration of 2 g/kg glucose (i.p.). Capillary blood drops were collected by tail-cut method immediately before (time 0) and 15, 30, 60, and 120 min after injection for blood glucose measurement through glucometer (Accu-check Active, Roche Diagnostic, Germany). Similar procedure was carried out for intraperitoneal insulin tolerance test (*ip*ITT), excepting animals were fed and received 1 IU/kg insulin (Humulin R, Lilly, USA). Glucose disappearance rate (*K*_ITT_) was derived from *ip*ITT data and calculated as 0.693/t_1/2_, where t_1/2_ is the half time to reach maximum blood glucose decay.

### Insulin Resistance Assessment

Insulin resistance was inferred from the calculation of TyG Index (TyG = Ln[fasting triglycerides (mg/dL) × fasting glucose (mg/dL)/2]) (Simental-Mendia et al., 2008), homeostasis model assessment (HOMA) Index of IR (HOMA-IR = fasting insulin (μU/mL)/fasting glucose (mM)/22.5) and HOMA Index of β-cell function [HOMA-B = 20 × fasting insulin (μU/ml)/fasting glucose (mM) – 3.5] (Matthews et al., 1985).

### Pancreatic Islet Isolation and Static Insulin Secretion Assays

Pancreatic islets from all groups were isolated by collagenase type V (0.8 mg/mL, Sigma–Aldrich, USA) digestion, as previously described (Bordin et al., 1995). For static insulin secretion assays, at least four groups of four islets per animal were pre-incubated in 24-wells plates for 45 min at 37°C in 1 mL Krebs-Hepes buffer (115 mM NaCl, 5 mM KCl, 2.6 mM CaCl_2_, 1 mM MgCl_2_, 10 mM NaHCO_3_, 15 mM HEPES, supplemented with 5.6 mM glucose, 3 g/L BSA) under controlled environment (95% O_2_ + 5% CO_2_). Afterward, incubation medium was replaced with fresh buffer containing eather 5.6 or 16.7 mM glucose and incubated for one additional hour. In another set of experiments, pancreatic islets from 90-days-old lean not-treated rats were incubated in eather 5.6 or 16.7 mM glucose Krebs-Hepes buffer containing HESc in the concentrations of 10, 30, 100, 300, and 1000 μg/mL for 1 h under same conditions. At the end, plates were cooled on ice bath and supernatants collected and appropriately stored at -20°C for posterior measurement of insulin concentrations by RIA, as earlier described.

### INS-1E β-Cell Line Culture and Insulin Secretion

INS-1E β-cells, generously donated by Dr. Claes Wollheim (University of Geneva, Geneva, Switzerland), were cultured in a humidified atmosphere (95%), containing 5% CO_2_ in complete medium RPMI 1640 (Sigma–Aldrich, Canada) supplemented with 5% inactivated fetal calf serum (Hyclone, Logan, UT), 1 mM sodium pyruvate, 50 mM 2-mercaptoethanol, 1 mM L-glutamine, 10 mM HEPES, 1 U/mL of penicillin and 1 mg/mL streptomycin (Sigma–Aldrich). To examine the effects of HESc (freshly diluted in water and filtered through 0.22 μm mesh) on insulin secretion, INS-1E cells were seeded in 96 wells plate (2 × 10^5^ cells/well) 24-h before the addition of HESc (100 or 1000 μg/mL) to culture medium for 4 h. Subsequently, culture medium was washed out with PBS (2×) and replaced by Krebs-Ringer Bicarbonate HEPES buffer (KRBH = 135 mM NaCl, 3.6 mM KCl, 5 mM NaHCO_3_, 0.5 mM NaH_2_PO_4_, 0.5 mM MgCl_2_, 1.5 mM CaCl_2_, and 10 mM HEPES, 0.1% BSA, pH 7.4) without glucose for 1-h. Then, KRBH was added 2.8 mM glucose for 30 min previous to the incubation with KRBH containing either 3.3 or 16.7 mM glucose for 2 h under the same atmosphere. Afterwhich, supernatants were collected and stored at -20°C for later insulin measurement with ultra-sensitive rat insulin ELISA kit (Crystal Chem Inc, USA) according to manufacturer’s instructions.

### Statistical Analysis

Results are expressed as mean ± SEM (*n* = 6–8 per group). Shapiro–Wilk test was applied for normality assuring. Intra- and inter-group comparisons were carried out by one-way ANOVA and multiple comparisons were obtained by Newman–Keuls post-test for a significance level of 5% (*p* < 0.05).

## Author Contributions

JS and LF maintained and treated animals, performed animal experiments, analyzed, and discussed data; VC performed experiments and analyzed data from phytochemical characterization of the extract; RG performed and analyzed data from cell culture experiments; KS maintained and treated animals, helped experimental procedures; LG performed and analyzed RIA measurements; DS and AH supervised execution of cell culture experiments, helped analyzed data and critically reviewed the manuscript; RD supervised HPLC-MS/MS experiments, analyzed and discussed data; EC supported RIA measurements, helped discussing data and critically reviewed the manuscript; AC supervised study execution, performed animal experiments and discussed data; AP conceived the study, supervised execution, analyzed and discussed data, drafted the manuscript. All authors read and approved manuscript final format.

## Conflict of Interest Statement

The authors declare that the research was conducted in the absence of any commercial or financial relationships that could be construed as a potential conflict of interest. The reviewer MH and handling Editor declared their shared affiliation, and the handling Editor states that the process nevertheless met the standards of a fair and objective review.
