# Supplementary material for: Polyphenol-Rich Extract of Syzygium cumini Leaf Dually Improves Peripheral Insulin Sensitivity and Pancreatic Islet Function in Monosodium L-Glutamate-Induced Obese Rats
Source: Front Pharmacol. 2016 Mar 10;7:48. doi: 10.3389/fphar.2016.00048 (PMC4785152; doi:10.3389/fphar.2016.00048)
Supplement: Supplementary file 1 [file Data_Sheet_1.PDF]

Mass spectra of Hydroethanolic Extract of *S. cumini* leaves (HESc)

Mass espectra obtained from the fragmentation of compounds (compounds 1-15 numbered in the chromatogram and available in table 1). (A) MS^1 spectrum containing the molecular ion of the phenolic compound; (B) MS^2 spectrum containing fragment ions of molecular ion. These data were used to tentative identification of polyphenolic compounds.

Compound 1

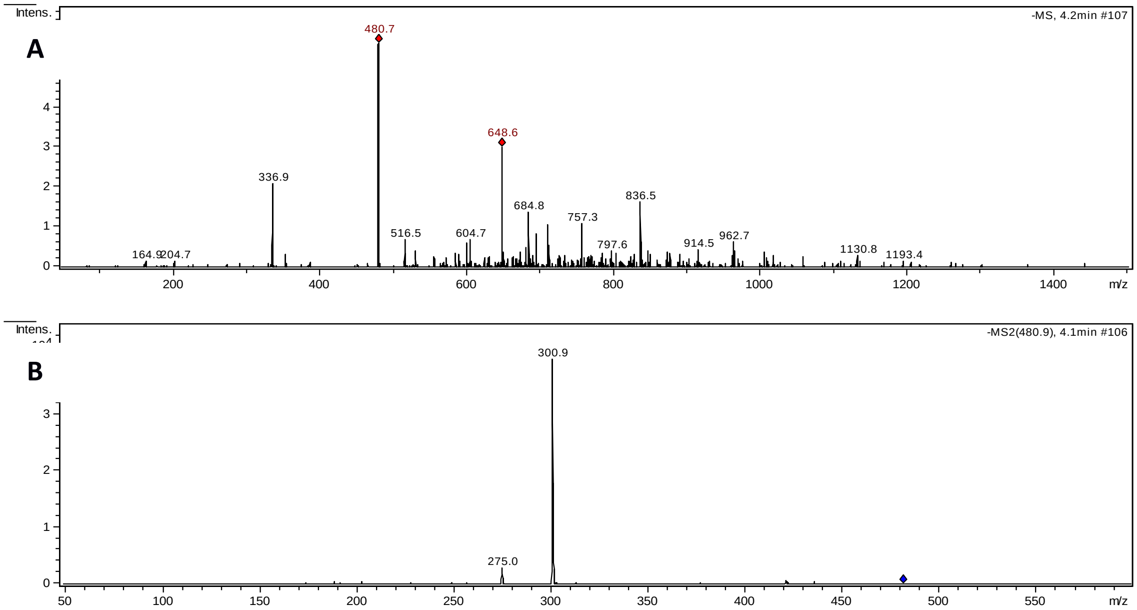

Compound 2

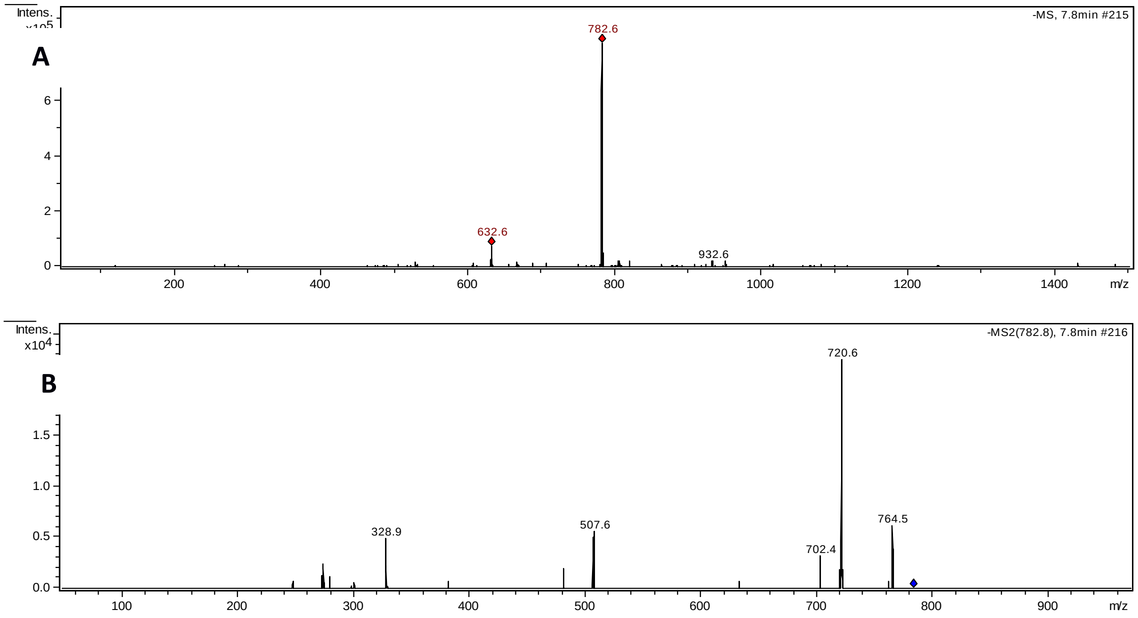

Compound 3

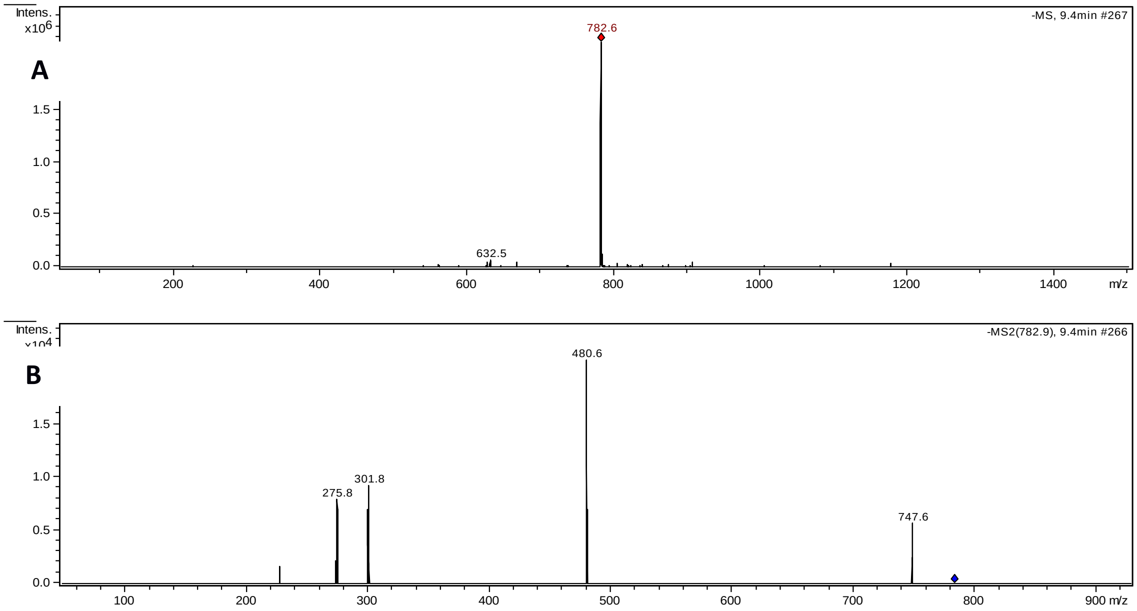

Compound 4

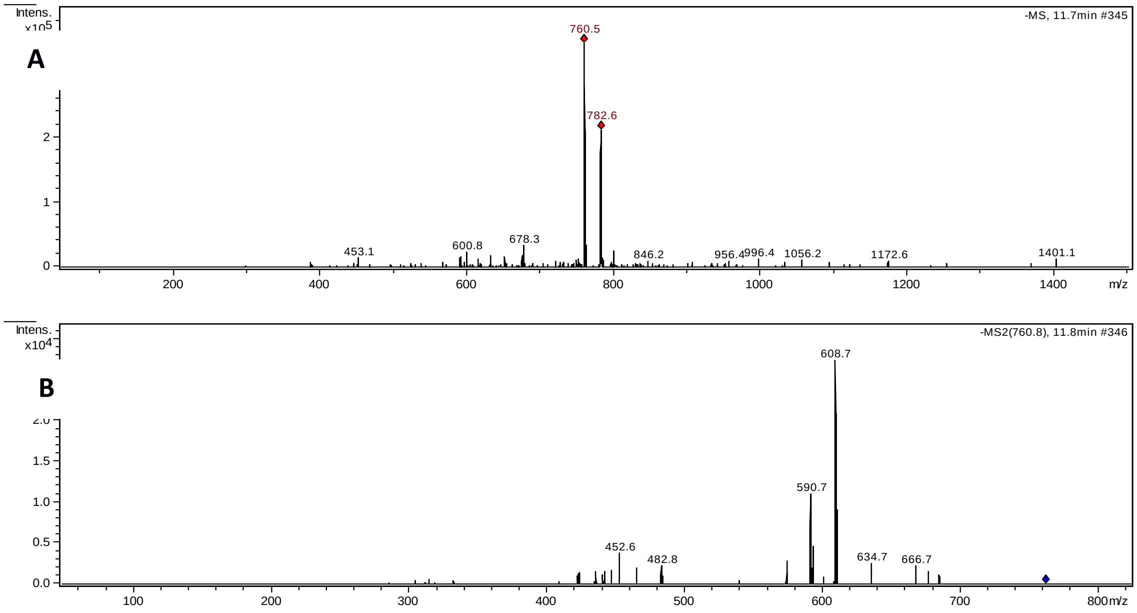

Compound 5

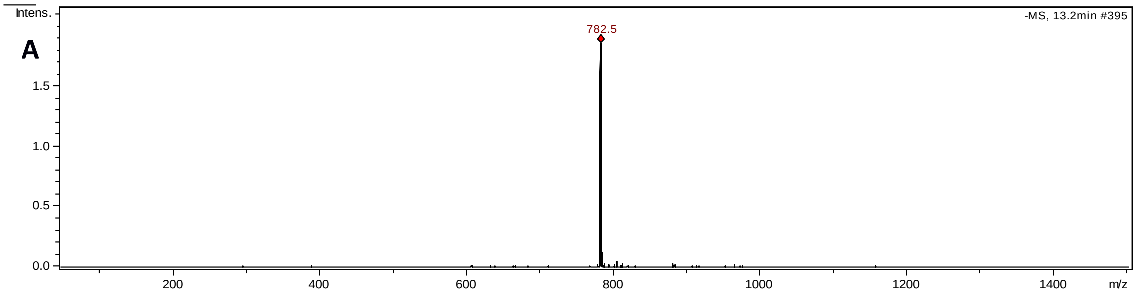

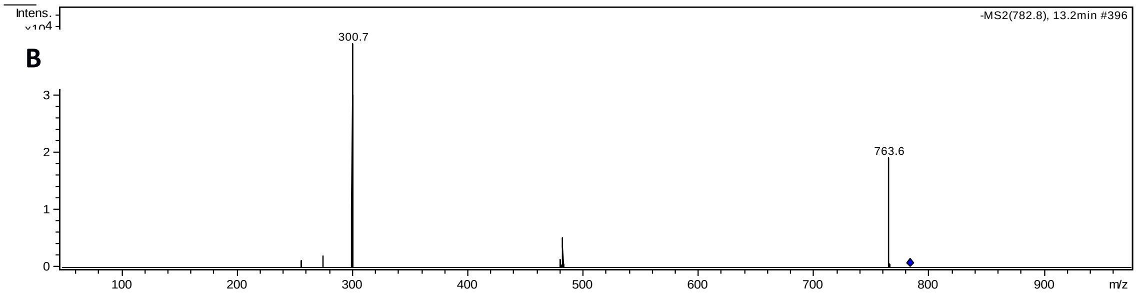

Compound 6

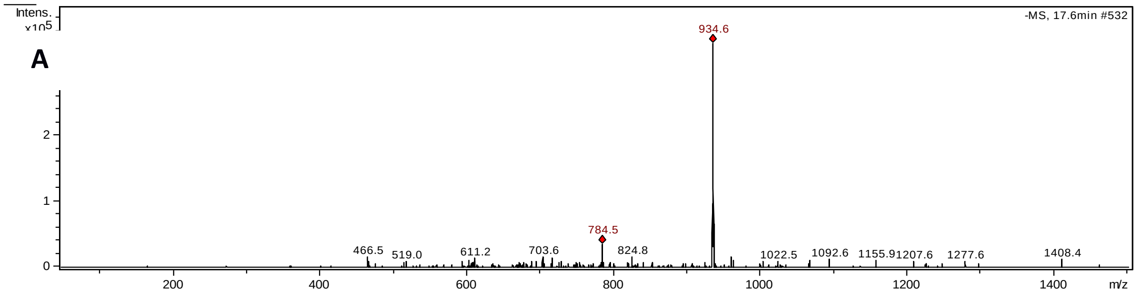

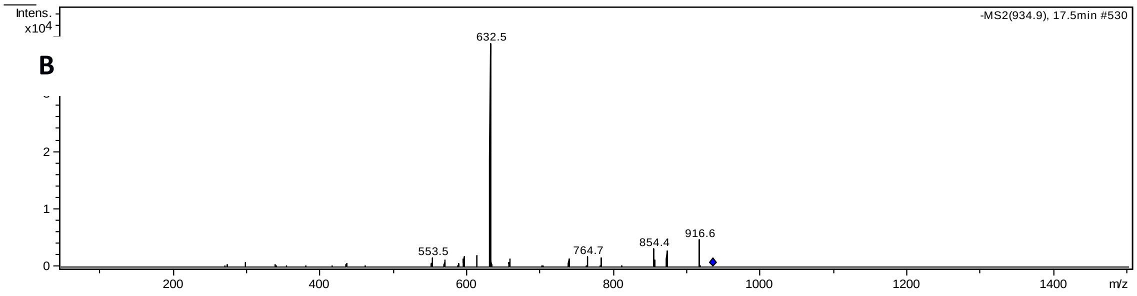

Compound 7

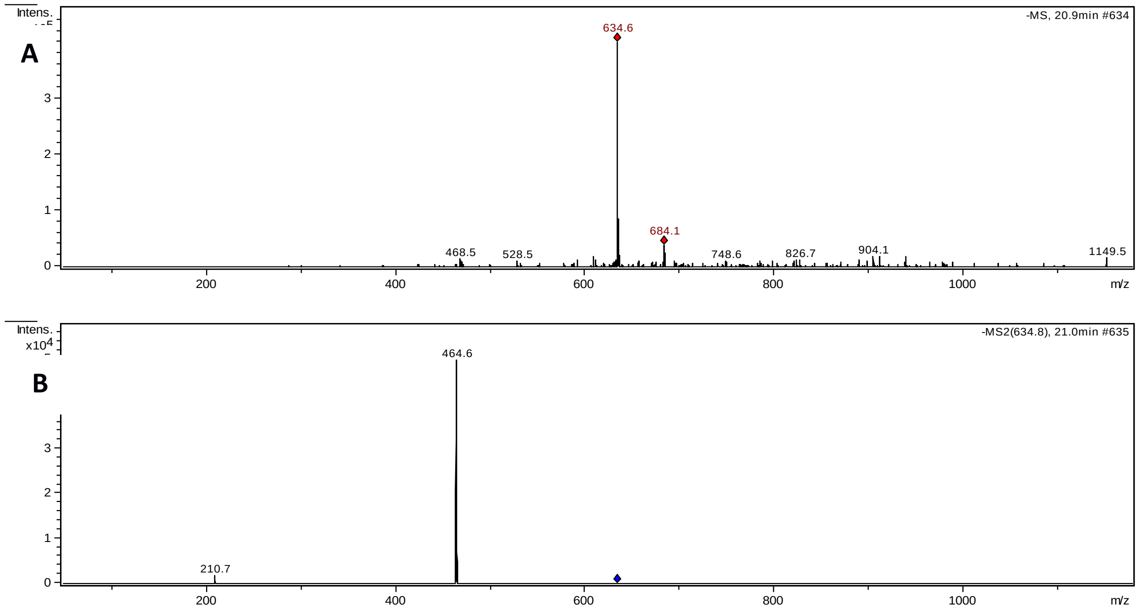

Compound 8

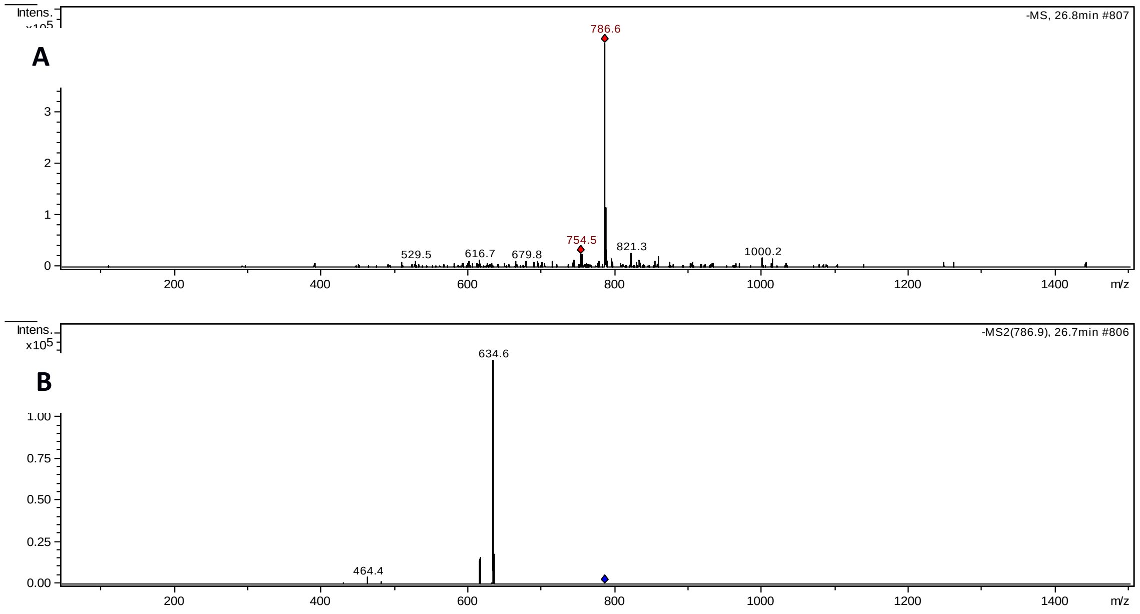

Compound 9

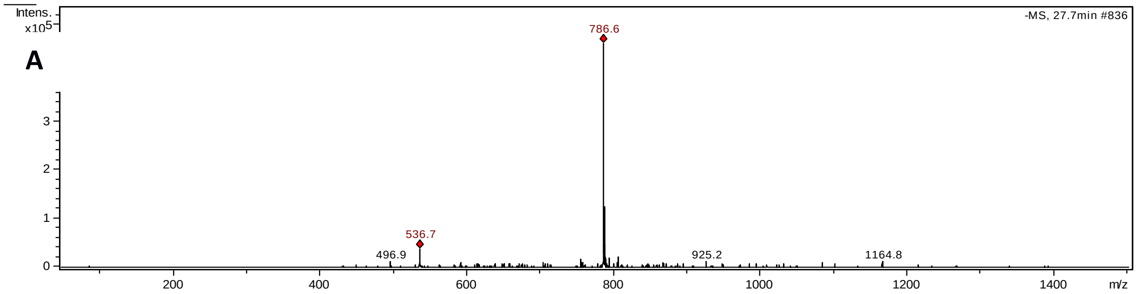

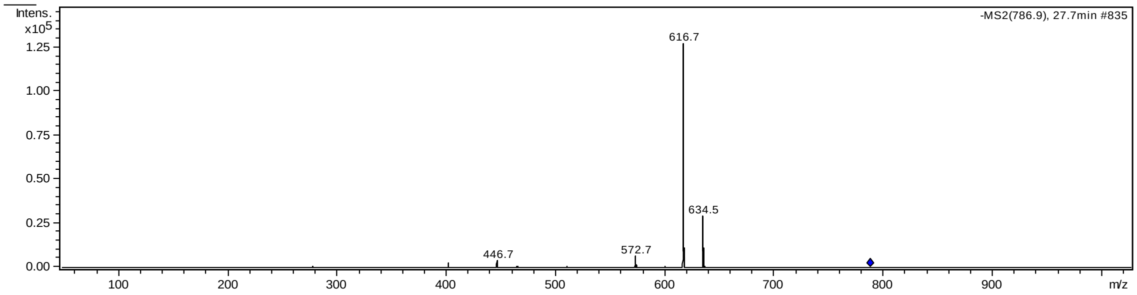

Compound 10

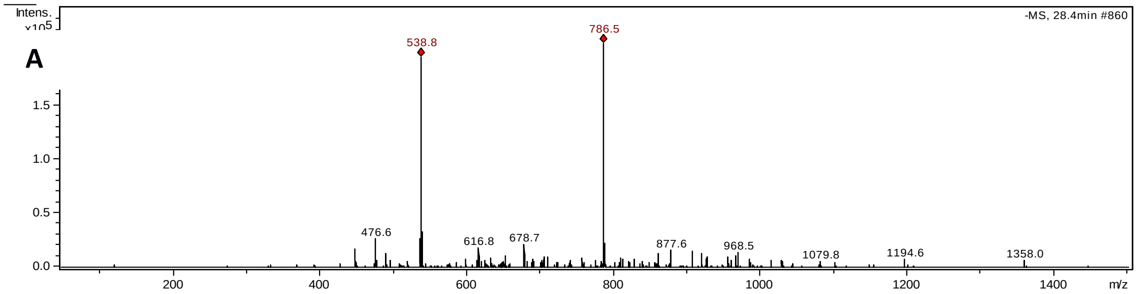

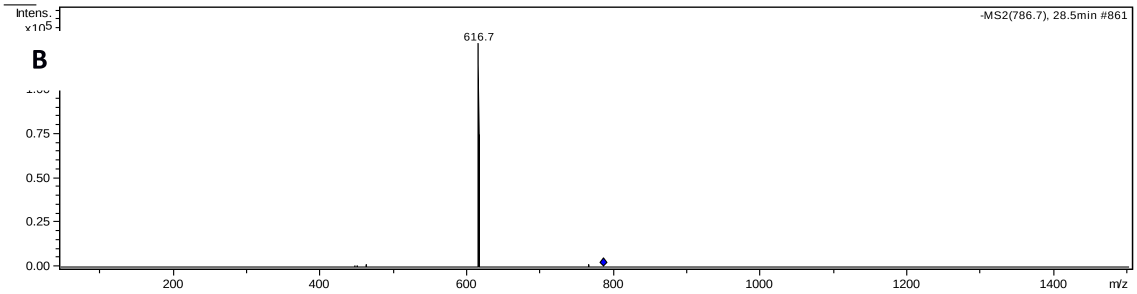

Compound 11

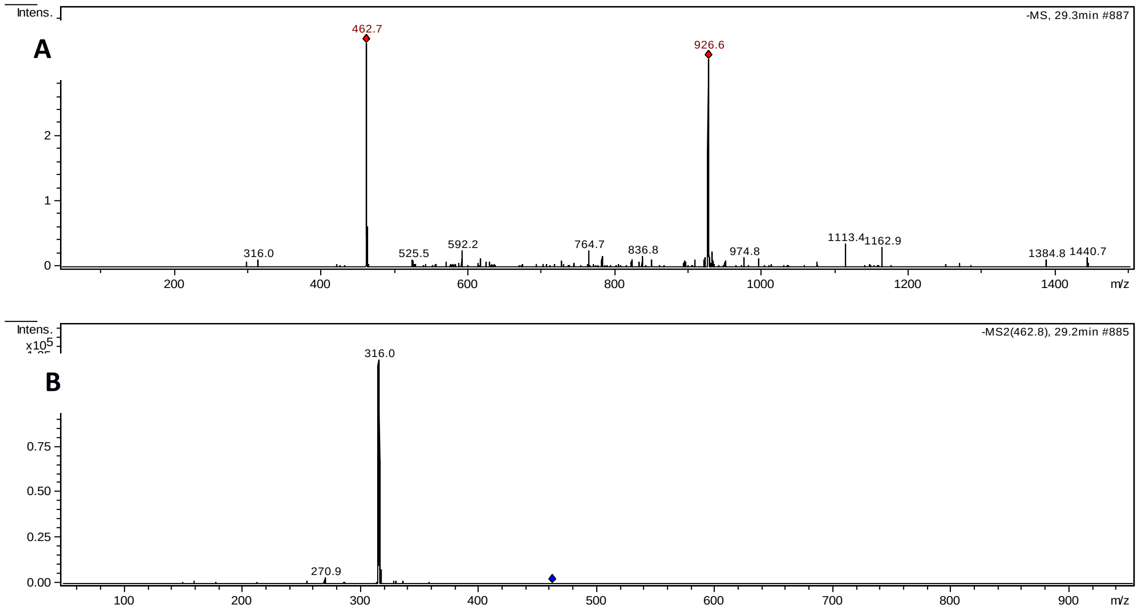

Compound 12

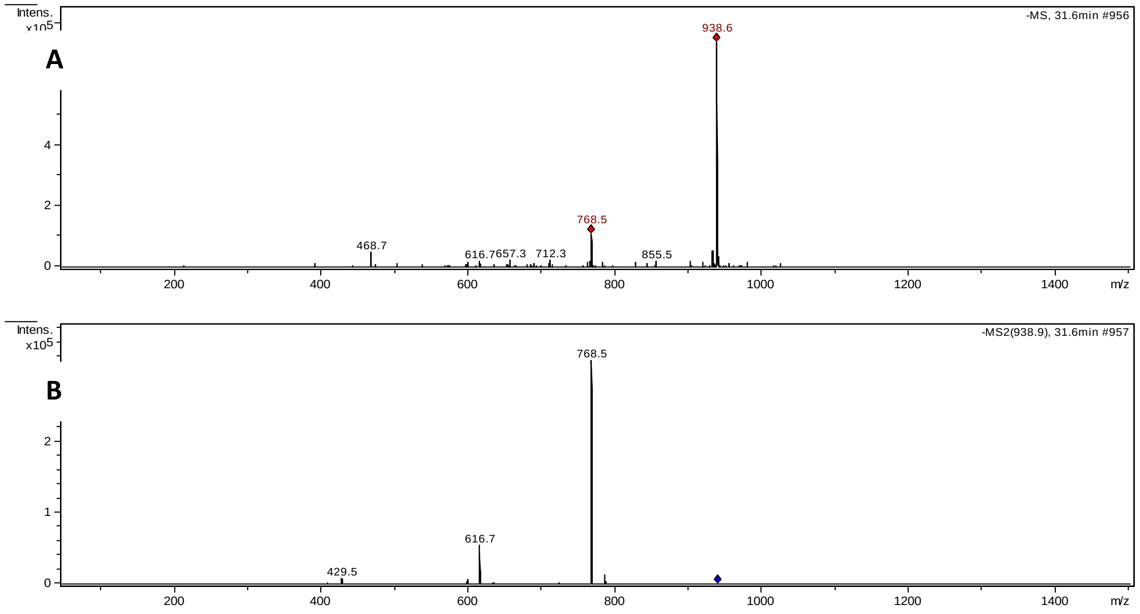

Compound 13

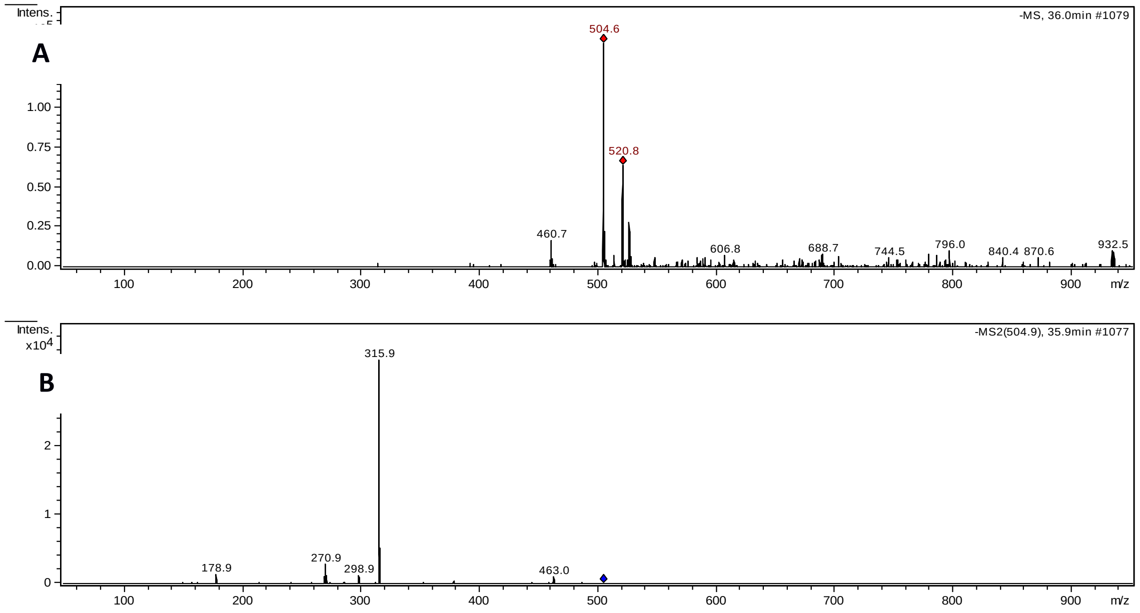

Compound 14

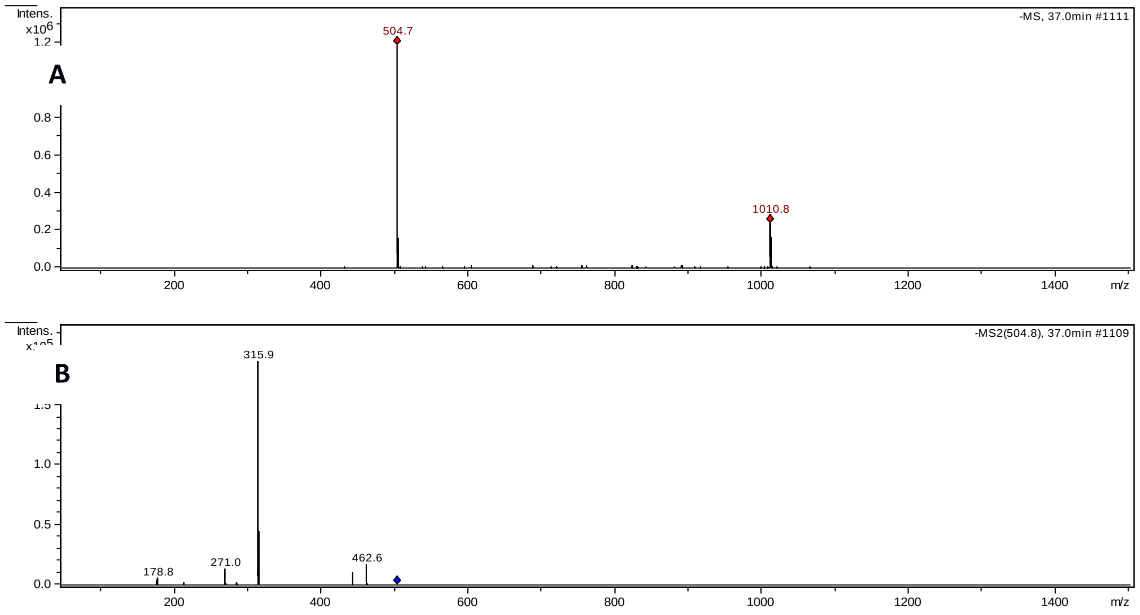

Compound 15

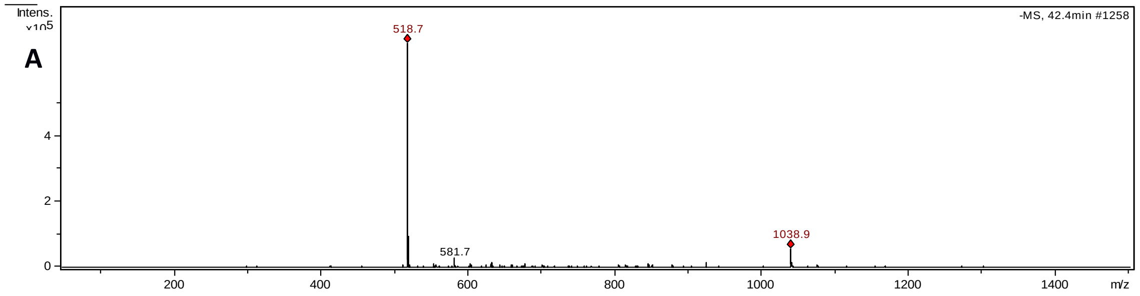
Intens.
x10^5
A
518.7
1038.9
581.7
-MS, 42.4min #1258
4
2
0
200
400
600
800
1000
1200
1400
m/z

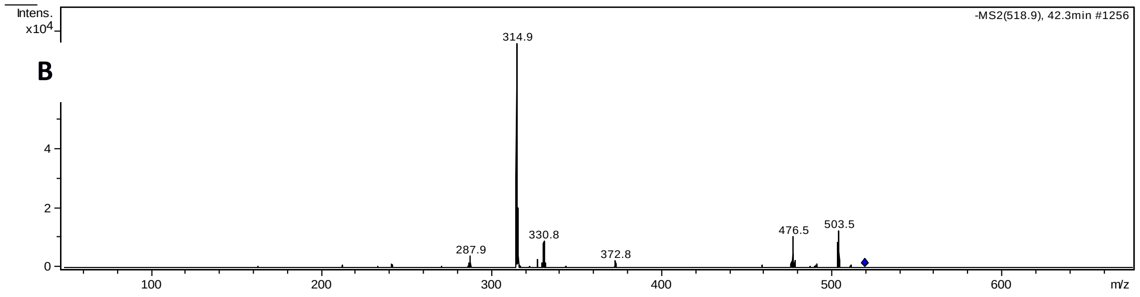
Intens.
x10^4
B
314.9
287.9
330.8
372.8
476.5
503.5
-MS2(518.9), 42.3min #1256
4
2
0
100
200
300
400
500
600
m/z
